# Supplementary material for: Khdc3 Regulates Metabolism Across Generations in a DNA-Independent Manner
Source: bioRxiv. 2024 Mar 1:2024.02.27.582278. Preprint. [Version 1] doi: 10.1101/2024.02.27.582278 (PMC10925209; doi:10.1101/2024.02.27.582278)
Supplement: Supplement 1 [file NIHPP2024.02.27.582278v1-supplement-1.pdf]

bioRxiv preprint doi: https://doi.org/10.1101/2024.02.27.582278; this version posted March 1, 2024. The copyright holder for this preprint (which was not certified by peer review) is the author/funder, who has granted bioRxiv a license to display the preprint in perpetuity. It is made available under aCC-BY 4.0 International license.

Additional Information

Supplementary Information is available for this paper.

Supplemental Material

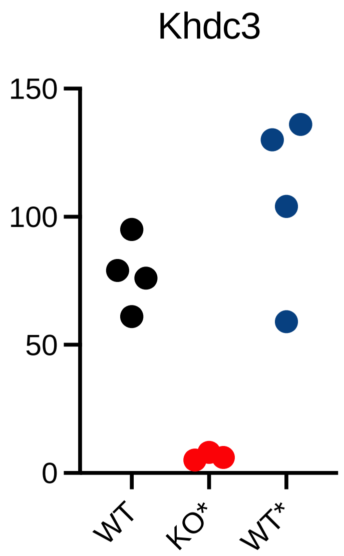

Supplemental Figure 1.

Khdc3 expression in ovaries of WT, KO*, and WT* mice, from RNA-Seq.

Supplemental Table 1: Primer sequences used for qPCR

Gene	Primer Sequence
**YWHAZ**	Forward primer: 5’ CAGAAGACGGAAGGTGCTGAGA 3’<br>Reverse primer: 5’ CTTTCTGGTTGCGAAGCATTGGG 3’
**Cyp17a1**	Forward primer: 5’ACTGCAGTGATTGTCGGTCA 3’<br>Reverse primer: 5’ CTAGAGTCACCATCTGGGGC 3’
**2610507I01Rik**	Forward primer: 5’ GGATCTGATAGTCGCCCGTG 3’<br>Reverse primer: 5’ TCGCAAGAGTTCCCTGCTTT 3’
